# Dietary mineral intake and lung cancer risk: the Rotterdam Study

**DOI:** 10.1007/s00394-016-1210-4

**Published:** 2016-04-12

**Authors:** Taulant Muka, Bledar Kraja, Rikje Ruiter, Lies Lahousse, Catherine E. de Keyser, Albert Hofman, Oscar H. Franco, Guy Brusselle, Bruno H. Stricker, Jessica C. Kiefte-de Jong

**Affiliations:** 1 Department of Epidemiology, Erasmus Medical Center, PO Box 2040, 3000 CA Rotterdam, The Netherlands; 2 Department of Biomedical Sciences, Faculty of Medicine, University of Medicine, Tirana, Albania; 3 University Clinic of Gastrohepatology, University Hospital Center Mother Teresa, Tirana, Albania; 4 Department of Internal Medicine, Groene Hart Hospital, Gouda, The Netherlands; 5 Department of Respiratory Medicine, Ghent University Hospital, Ghent, Belgium; 6 Health Care Inspectorate, The Hague, The Netherlands; 7 Leiden University College, The Hague, The Netherlands

**Keywords:** Zinc, Iron, Calcium, Copper, Magnesium, Selenium, Lung cancer

## Abstract

**Objective:**

Limited data are available on the role of mineral intake in the development of lung cancer (LC). We investigated whether dietary calcium, copper, iron, magnesium, selenium and zinc intake were associated with LC risk.

**Methods:**

We analyzed data from 5435 participants of the Rotterdam Study, a prospective population-based cohort study among subjects aged 55 years and older. At baseline (1990–1993), diet was measured by a validated food frequency questionnaire. LC events were diagnosed on the basis of pathology data and medical records. Hazard ratios (HRs) on LC for energy-adjusted mineral intake were calculated using Cox regression models while adjusting for potential confounders.

**Results:**

During a follow-up period of 22 years, we identified 211 incident cases of LC. A higher zinc intake was associated with 42 % reduction in risk of LC (top tertile vs. first tertile: HR 0.58, 95 % CI 0.35; 0.94, *P*-for trend = 0.039). Similarly, high intake of iron was associated with reduced risk of LC (top tertile vs. first tertile: HR 0.58, 95 % CI 0.37; 0.92, *P*-for trend = 0.021). There was no association between dietary intake of calcium, copper, magnesium and selenium and LC risk.

**Conclusions:**

Our results suggest that dietary zinc and iron intake are associated with reduced risk of LC. No evidence was found for an association between calcium, copper, magnesium and selenium intake and LC risk.

**Electronic supplementary material:**

The online version of this article (doi:10.1007/s00394-016-1210-4) contains supplementary material, which is available to authorized users.

## Introduction

Lung cancer is the leading cause of cancer mortality worldwide and incurs the highest economic burden of all cancers [1, 2]. Lung cancer is a disease for which environmental factors (e.g., smoking, gender, asbestosis and pollution) play an important role [3]. Besides smoking, emerging evidence also suggest that dietary factors may have an impact on the risk of lung cancer [3]. To date, limited evidence exists on the role of dietary mineral intake, such as calcium, copper, iron, magnesium, selenium and zinc in the development of lung cancer [4–8]. Copper, magnesium, selenium and zinc are essential dietary minerals for maintaining the integrity of DNA by preventing oxidative DNA damage [9–12]. On the other hand, iron deficiency, as well as iron overload, may lead to oxidative DNA damage [13]. Evidence from in vitro and experimental studies suggests that DNA damage and defects in DNA repair mechanisms can predispose to cancer development [14]. Likewise, epidemiological studies have shown that DNA repair capacity is associated with increased lung cancer risk [3, 15, 16]. Calcium is another important mineral involved in processes of cell proliferation and carcinogenesis through cell signaling and cell cycle regulation [17]. However, previous studies evaluating the relationship between dietary mineral intake and lung cancer risk have shown inconsistent results [4, 5, 18]. Moreover, the most recent report from the World Cancer Research Fund concluded that current evidence is still insufficient to allow any dietary recommendations for calcium, copper, iron, magnesium, selenium and zinc to reduce the risk of lung cancer [3].

The present study aimed to investigate whether dietary intake of these minerals was associated with lung cancer risk in a prospective population-based cohort study in the Netherlands.

## Subjects and methods

### Study population

**Fig. 1:**
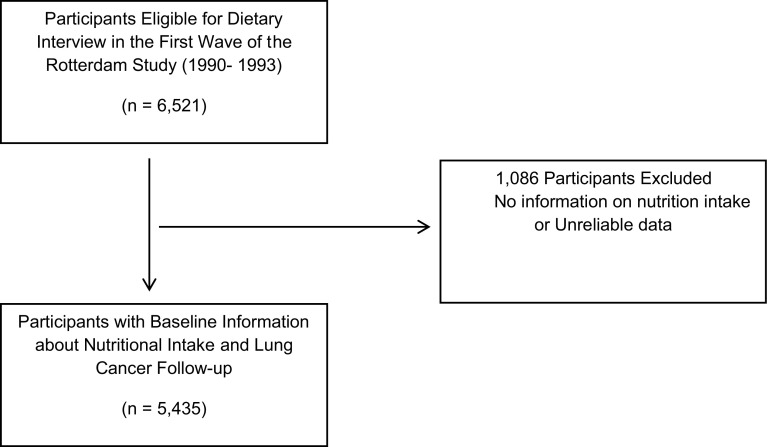
Flow chart of participants in the study, the Rotterdam Study, 1989–1993

This study was embedded in the first cohort of The Rotterdam Study (RS), a prospective population-based cohort study that started in 1990 with the aim to examine the frequency and determinants of diseases in elderly [19, 20]. The rationale and design of the RS are described elsewhere [19, 20]. Trained research assistants collected data on current health status, use of medication, and medical history, lifestyle, and risk indicators for chronic diseases during an extensive home interview at baseline (1990–1993). Subsequently, the participants visited the study center for detailed clinical examinations and assessment of diet. Follow-up visits were held every 3–5 years [19]. The Medical Ethics Committee of the Erasmus Medical Center approved the study, and written consent was obtained from all participants. Out of the initial cohort (*N* = 7983), 6521 who visited the study center at baseline were eligible for a dietary interview. We excluded 1086 (17 %) participants without reliable dietary data (i.e., no dietary data were collected from nursing home residents, and dietary data were considered as unreliable by the dietician, e.g., when subjects did not fully cooperate during the dietary interview). Therefore, 5435 participants were included in the final analysis (Fig. 1).

### Assessment of dietary intake

During the first home interview (1990–1993), participants completed a checklist that included foods and drinks they had consumed at least twice a month during the preceding year, as well as dietary habits, use of supplements, and prescribed diets. Next, during their visit to the research center, they underwent a standardized interview with a dietitian based on the checklist, using a 170-item semiquantitative food frequency questionnaire [21]. A validation study comparing this questionnaire with a 2-week food diary demonstrated reproducible and valid estimates; Pearson’s correlation after adjustment for age, sex, energy and within-person variation were between 0.44 and 0.85 for macro- and micronutrients as described in detail previously [21]. Specifically, adjusted Pearson’s correlation coefficient was 0.44 for iron, 0.54 for zinc, 0.71 for selenium and 0.72 for calcium, while the result for copper was not reported. Energy and nutrient intake were estimated based on frequency of consumption and standardized portion sizes and using Dutch food composition table of 1993 and 1998 [22, 23] (heme and non-heme iron was not included in the 1993 table; therefore, the 1998 table was used). Data on zinc, iron (total iron, heme and non-heme iron), magnesium, selenium, copper and calcium were available.

### Follow-up and case identification

Follow-up of each participant began on the date at which baseline dietary intake was assessed (1989–1993) and ended on the date of lung cancer diagnosis, death, loss to follow-up or administrative censoring (31 December 2011), whichever occurred first. Two research physicians independently assessed the diagnoses and the type of lung cancer on the basis of pathology data and medical records. All events were pathology proved and classified according to the International Classification of Disease (ICD), tenth edition.

### Covariates assessment

The following covariates measured at baseline were considered as potential confounders: age; sex; smoking status (never smokers, former smokers <15 pack-years, former smoker ≥15 pack-years, current smoker <27.5 pack-years, current smoker ≥27.5 pack-years), use of alcohol (grams of ethanol per day); education level (low: primary education, intermediate: secondary general or vocational education, or high: higher vocational education or university); income level (low, intermediate or high), unprocessed red meat intake; processed red meat intake; total energy intake; use of hormone replacement therapy and use of minerals supplements (self-reported at baseline home interview); diabetes mellitus (defined as a fasting serum glucose level ≥11 mmol/L or the use of glucose lowering drugs); family history of cancer and Dutch Healthy Diet-index (DHD-index) (to take into account the overall dietary quality, DHD-index represents compliance to the Dutch Guidelines for a Healthy Diet as assessed from the FFQ at baseline [24]). Baseline physical height and body weight were measured with the participants standing without shoes and heavy outer garments. Body mass index, calculated as weight divided by height squared (kg/m^2^), was also considered as confounder. Physical activity, assessed in the third round (1997–1999) of the Rotterdam Study by an adapted version of the Zutphen Physical Activity Questionnaire and the LASA Physical Activity Questionnaire [25, 26], was considered as confounder as well.

### Statistical analysis

Age- and sex-adjusted (crude) and multivariable Cox proportional hazards models were computed to estimate hazard ratios (HRs) and 95 % confidence intervals (CIs) for each tertile of mineral intake compared with the lowest tertile as the reference category. All dietary intake data were energy-adjusted by using the residual method [27]. In addition to age and gender, multivariable models were further adjusted for body mass index (BMI) (continuous), smoking (never smokers, former smokers <15 pack-years, former smoker ≥15 pack-years, current smoker <27.5 pack-years, current smoker ≥27.5 pack-years), alcohol intake (continuous), education level (low, intermediate, high), income (low, intermediate, high), energy-adjusted unprocessed red meat (continuous), energy-adjusted processed red meat (continuous), total energy intake (continuous), use of hormone replacement therapy (HRT) (yes vs. no), prevalent diabetes mellitus (yes vs. no), family history of cancer (yes vs. no), Dutch Healthy Diet-index (DHD-index) (continuous), minerals supplemental use (yes vs. no) and physical activity (continuous). Due to significant correlations between dietary minerals (Supplemental Table 1), we mutually adjusted for the other selected minerals in the multivariable model. To examine whether the association between dietary minerals intake and lung cancer differed by sex and smoking status, we tested for statistical interaction by adding a multiplicative interaction term (mineral intake x sex/smoking) to the age- and gender-adjusted model. In case of significant effect modification, results were presented stratified by sex or by smoking status (former/current smokers and ever smokers). All the values presented were two-sided, and *P* < 0.05 was considered statistically significant. To adjust for potential bias associated with missing data, a multiple imputation procedure was used for missing covariates (*N* = 5 imputations). For the pooled regression coefficients (*β*) and 95 % CIs, we used Rubin’s method [28].

Since high correlations between zinc and iron intake with red and processed meat were present (Table 2) but red meat intake has also been reported to be associated with an increased risk of lung cancer [29], we did a sensitivity analysis by excluding red and processed meat from the multivariable model. To look if different sources of iron had different role, we further examined the association between heme and non-heme iron with lung cancer risk. To evaluate whether change in diet due to early signs of lung cancer may have influenced our results and whether minerals supplementation could have influenced our results, we repeated the analysis by excluding subjects who developed lung cancer in the first 2 years of follow-up and by excluding subjects that used any mineral supplementation. To investigate whether the results are different for the different subtypes of lung cancer, we examined the association of mineral intake with non-small cell lung cancer and other histologic subtypes. Also, a sensitivity analysis was preformed substituting smoking status categorized in five categories with smoking status categorizes into ever/formers versus current smokers. Furthermore, we restricted the main analysis only to subjects who reported to be ever or former smokers. All the *P* values presented were two-sided, and *P* < 0.05 was considered statistically significant. All analyses were done using SPSS statistical software (SPSS, version 21.0; SPSS Inc, Chicago, Illinois).

## Results

During a mean follow-up time of 15.2 years, a total of 211 subjects developed lung cancer (128 cases in men and 83 cases in women). Baseline characteristics are listed in Table 1. Mean total dietary intake of minerals was as follows: zinc: 10.6 ± 2.7 mg/day; iron: 11.9 ± 2.9 mg/day; magnesium: 306.2 ± 74.8 mg/day; selenium: 32.7 ± 10.5 µg/day: copper: 1.2 ± 0.5 mg/day; calcium: 1125.5 ± 402.8 mg/day.

**Table 1:** Selected characteristic of study participants (*n* = 5435)

Characteristics	Value
Age, years	70.5 ± 5.9^a^
Female, *n* (%)	59.1 (3210)
Smoking status, *n* (%)	
Never	1838 (33.8)
Former smokers	2334 (42.9)
Current smokers	1263 (23.3)
Physical activity (min/week)	2550.50 ± 1133.1
Alcohol intake, g/day	3.44 (14.7)^b^
Education level, *n* (%)	
Low	2830 (52.1)
Medium	2140 (39.4)
High	465 (8.6)
Income, *n* (%)	
Low	1265 (23.3)
Medium	2415 (44.4)
High	1755 (32.3)
BMI (kg/m^2^)	26.33 ± 3.64
DHD-index	48.24 ± 10.10
Unprocessed red meat (g/day)	74.37 ± 47.80
Processed red meat (g/day)	21.94 ± 18.75
Total energy intake (kcal/day)	1974.2 ± 502.6
Hormone replacement therapy *n* (%)	85 (1.6)
Diabetes mellitus	517 (9.5)
Mineral supplemental use, *n* (%)	101 (1.9)
Family history of cancer, *n* (%)	2804 (51.6)
Zinc intake (mg/day)	10.6 ± 2.7
Iron intake (mg/day)	11.9 ± 2.9
Heme iron intake (mg/day)	2.3 ± 0.98
Non-heme iron intake (mg/day)	8.8 ± 2.4
Magnesium intake (mg/day)	306.2 ± 74.8
Selenium intake (µg/day)	32.7 ± 10.5
Copper (mg/day)	1.2 ± 0.5
Calcium intake (mg/day)	1125.5 ± 402.8

*DHD-index* Dutch healthy diet-index

^a^Mean ± SD (all such values)

^b^Median; interquartile range in parentheses (all such values)

Specific dietary food intakes that significantly contributed to zinc, iron, magnesium, selenium, copper and calcium intake are shown in Table 2. The Pearson (*r*) correlations of 0.51, 0.45 and 0.42 were found between zinc intake and, respectively, processed and red meat, total dairy and wholegrain (Table 2). The correlations of iron with the specific dietary foods intake (Table 2) showed the highest correlations for wholegrain and processed and red meat (*r* = 0.51, 0.45, respectively). The main food contributor for magnesium, selenium, copper and calcium were wholegrain (*r* = 0.57), fish (*r* = 0.62), processed and red meat (*r* = 0.28) and total dairy, respectively (*r* = 0.86). The overall variance explained by these food items for zinc, iron, magnesium, selenium, copper and calcium intake varied from 27 % for copper to 80 % for calcium (Table 2).

**Table 2:** Correlation coefficients between dietary mineral intake and their dietary food sources

	Zinc (g/day) Pearson’s correlation *(r)*	Iron (g/day) Pearson’s correlation *(r)*	Magnesium (g/day) Pearson’s correlation *(r)*	Selenium (g/day) Pearson’s correlation *(r)*	Copper (g/day) Pearson’s correlation *(r)*	Calcium (g/day) Pearson’s correlation *(r)*
Vegetable oils (g/day)	0.06	0.10	0.09	0.12	0.06	–
Butter, margarines and hard frying fats (g/day)	0.25	0.34	0.26	0.23	0.16	0.10
Fish (g/day)	0.10	0.06	0.10	0.62	0.08	0.07
Poultry(g/day)	0.10	0.08	0.10	0.18	0.13	–
Processed and red meat (g/day)	0.51	0.45	0.17	0.23	0.28	-0.05
Sweet desserts and confectionary (g/day)	0.09	0.20	0.18	0.16	0.14	0.07
Chips (g/day)	–	0.03	–	–	0.04	–
Wholegrain (g/day)	0.42	0.51	0.57	0.23	0.21	0.30
Total dairy^a^ (g/day)	0.45	0.06	0.49	0.35	0.12	0.86
Fruit (g/day)	0.11	0.16	0.28	0.15	0.20	0.20
Pulses (g/day)	0.09	0.21	0.10	0.06	0.08	0.04
Eggs (g/day)	0.13	0.15	0.09	0.30	0.15	–
Nuts (g/day)	0.23	0.22	0.31	0.19	0.25	0.05

Overall variance explained by these food items: 70 % for zinc intake; 57 % for iron intake; 72 % for magnesium; 75 % for selenium; 27 % for copper; 80 % for calcium

^a^Including cheese

“-” *p* value >0.05; otherwise, *p* value <0.05

### Dietary intake of minerals and lung cancer risk

A higher zinc intake was associated with 42 % reduction in the risk of lung cancer (top tertile vs. first tertile: HR 0.58, 95 % CI 0.35; 0.94, *P*-for trend = 0.039, Table 3) after adjustment for age, sex, alcohol intake, BMI, smoking status, physical activity, DHD-index, processed and unprocessed red meat, energy intake, HRT, presence of diabetes mellitus, socioeconomic status, family history of cancer and dietary intake of other minerals. Similarly, high intake of iron was associated with reduced risk for lung cancer (in multivariable adjusted models: top tertile vs. first tertile: HR 0.58, 95 % CI 0.37; 0.92, *P*-for trend = 0.021, Table 3). No significant hazard ratios were found in the fully adjusted models for the association between dietary magnesium, calcium selenium and copper intake and the risk of lung cancer (Table 3).

**Table 3:** HRs of lung cancer by categories of dietary mineral intake

*N* = 5435	Tertile 1	Tertile 2	Tertile 3	Continuous	*P* trend
Dietary zinc intake					
Cases, *n*	83	77	51		
Model 1 HR, 95 % CI	1.00	0.93 (0.68, 1.27)	0.57 (0.40, 0.81)	0.89 (0.83, 0.96)	0.002
Model 2 HR, 95 % CI	1.00	1.02 (0.72, 1.46)	0.58 (0.35, 0.94)	0.88 (0.80, 0.98)	0.039
Dietary iron intake					
Cases, *n*	85	65	61		
Model 1 HR, 95 % CI	1.00	0.73 (0.53, 1.01)	0.60 (0.43, 0.84)	0.94 (0.89, 0.997)	0.003
Model 2 HR, 95 % CI	1.00	0.80 (0.56, 1.15)	0.58 (0.37, 0.92)	0.94 (0.86, 1.04)	0.021
Dietary magnesium Intake					
Cases, *n*	88	60	63		
Model 1 HR, 95 % CI	1.00	0.68 (0.49, 0.95)	0.66 (0.47, 0.91)	0.997 (0.994, 0.999)	0.011
Model 2 HR, 95 % CI	1.00	0.80 (0.57, 1.14)	0.84 (0.56, 1.27)	0.998 (0.995, 1.001)	0.38
Dietary selenium intake					
Cases, *n*	70	62	79		
Model 1 HR, 95 % CI	1.00	0.94 (0.67, 1.32)	1.16 (0.84, 1.61)	1.008 (0.993, 1.023)	0.35
Model 2 HR, 95 % CI	1.00	1.06 (0.74, 1.52)	1.39 (0.97, 1.99)	1.011 (0.996, 1.027)	0.07
Dietary copper intake					
Cases, *n*	75	67	69		
Model 1 HR, 95 % CI	1.00	0.95 (0.68, 1.33)	0.97 (0.69, 1.34)	1.17 (0.999, 1.37)	0.83
Model 2 HR, 95 % CI	1.00	1.33 (0.93, 1.91)	1.23 (0.84, 1.81)	1.20 (0.91, 1.06)	0.28
Dietary calcium intake					
Cases, *n*	90	64	57		
Model 1 HR, 95 % CI	1.00	0.79 (0.57, 1.08)	0.72 (0.51, 1.004)	1.000 (0.999, 1.000)	0.047
Model 2 HR, 95 % CI	1.00	0.89 (0.59, 1.33)	0.76 (0.40, 1.45)	0.996 (0.889, 1.115)	0.42

*HR* hazard ratio, *CI* confidence interval

Model 1: Adjusted for age and sex

Model 2: Model 1 + alcohol intake(continuous), body mass index (continuous), smoking status (never smokers, former smokers <15 pack-years, former smoker ≥15 pack-years, current smoker <27.5 pack-years, current smoker ≥27.5 pack-years), physical activity(continuous), Dutch healthy diet-index (continuous), dietary processed meat intake (continuous), dietary unprocessed red meat intake(continuous), total energy intake (continuous), hormone replacement therapy (yes vs. no), diabetes mellitus (yes vs. no), education status(low, intermediate, high), income status (low, intermediate, high), total energy adjusted sum of other minerals (excluding the mineral under investigation) (continuous) and family history of cancer (yes vs. no)

### Effect modification by gender and smoking

Significant interaction terms with gender were observed for dietary iron intake (*P*-*interaction* = 0.027) and copper (*P*-*interaction* = 0.029). After stratification by gender, the inverse association between dietary iron intake and lung cancer was only observed in men (top tertile vs. first tertile: HR 0.49, 95 % CI 0.28; 0.85, *P*-for trend = 0.016, Supplemental Table 2–3) but not in women (Supplemental Table 2-3). No consistent results were found for dietary copper in either gender (Supplemental Table 2–3). No statistically significant interactions were observed between dietary zinc intake (*P*-*interaction* = 0.49), magnesium (*P*-*interaction* = 0.42) or calcium intake (*P*-*interaction* = 0.34) and gender (Supplemental Table 2–3). Also, no significant interaction terms were observed for any of the minerals and smoking status (*P*-*interactions* varied from 0.16 to 0.97) (data not shown).

### Additional analysis

**Fig. 2:**
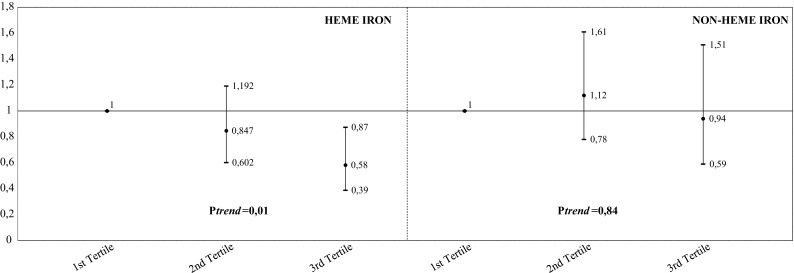
Multivariable HRs of lung cancer by categories of dietary heme iron and non-heme iron intake. HRs (95 % CI) were estimated by using Cox’s proportional hazard model adjusted for age, gender, alcohol intake (continuous), body mass index (continuous), smoking status (never smokers, former smokers <15 pack-years, former smoker ≥15 pack-years, current smoker <27.5 pack-years, current smoker ≥27.5 pack-years), physical activity (continuous), Dutch healthy diet-index (continuous), dietary processed meat intake (continuous), dietary unprocessed red meat intake (continuous), total energy intake (continuous), hormone replacement therapy (yes vs. no), diabetes mellitus (yes vs. no), education status (low, intermediate, high), income status (low, intermediate, high), total energy adjusted sum of other minerals (excluding the mineral under investigation) (continuous), family history of cancer (yes vs. no) and heme or non-heme iron (according to the exposure under investigation). *HR* hazard ratio, *CI* confidence interval

When the results were not adjusted for red meat, the inverse associations between zinc intake (top tertile vs. first tertile: HR 0.68, 95 % CI 0.44; 1.08, *P*-for trend = 0.12,) and iron intake (top tertile vs. first tertile: HR 0.65, 95 % CI 0.42; 1.02, *P*-for trend = 0.06,) and the risk of lung cancer were not significant. Heme iron intake was inversely associated with lung cancer risk (in multivariable adjusted models: top tertile vs. first tertile: HR 0.58, 95 % CI 0.39; 0.87, *P*-for trend = 0.01, Fig. 2), whereas no association was found between non-heme iron and lung cancer risk (in multivariable adjusted models: top tertile vs. first tertile: HR 0.94, 95 % CI 0.59; 1.51, *P*-for trend = 0.84, Fig. 2). Additional analyses showed that the association between heme iron intake and lung cancer risk was not significant when the results were not adjusted for red meat (top tertile vs. first tertile: HR 0.75, 95 % CI 0.53; 1.07, *P*-for trend = 0.11). Substitution of smoking status as five categories into smoking categorized as ever/formers vs. current smokers or restriction of the main analysis among subjects who were ever/former smokers (total number = 4172, number of lung cancer cases = 90) did not materially affect any of the associations (data not shown).

All associations that were statistically significant in the main analysis remained unchanged in terms of statistical significance in sensitivity analyses when excluding subjects who used mineral supplements (*n* = 101) (data not shown). Similarly, exclusion of lung cancer cases that occurred during the first 2 years of follow-up (*n* = 24) from our analysis did not substantially affect our results (Supplemental Table 4). Furthermore, the associations were not significantly different when the subtypes of lung cancer were examined separately (Supplemental Table 5–6).

## Discussion

In this prospective population-based cohort study, we found that dietary intake of zinc and iron was associated with a decreased risk of lung cancer. In contrast, we found no evidence that dietary intake of calcium, copper, magnesium and selenium was associated with lung cancer risk. Epidemiological studies evaluating the role of dietary mineral intake on lung cancer risk have reported inconsistent results [4–7, 18]. As a result, no consensus has been reached by the WCRF [3].

In our study, the highest tertile of zinc intake was associated with 42 % reduction in the risk for lung cancer, suggesting a beneficial role of dietary zinc intake on lung carcinogenesis. Similar to our findings, three large case–control studies reported an inverse association between zinc intake and lung cancer risk with risk reductions in the same magnitude [5, 6, 16]. Moreover, a recent observation from Hashemian et al. [30] showed that zinc intake can reduce the risk of esophageal cancer. However, findings from two prospective studies investigating the association between zinc intake and lung cancer were inconsistent; one prospective study based on 482,875 subjects with a mean follow-up of 7 years showed no beneficial role of dietary zinc intake on lung cancer [4], whereas another study conducted in 34,708 postmenopausal women with 16 years of follow-up showed that there was no association between zinc intake and lung cancer overall, but a high dietary zinc intake may decrease the risk of lung cancer among women who consume high-dose vitamin C supplements [7]. In contrast to our study, the previous two prospective studies did not adjust for red meat, which can explain the difference in the reported results. As shown in our sensitivity analysis, when the results were not adjusted for red meat, the beneficial inverse association between dietary zinc intake and lung cancer was not significant. These results may imply that zinc from food sources other than red meat may be protective against lung cancer. Several mechanisms may explain the beneficial role of zinc on lung carcinogenesis. Zinc has been shown to protect against free radicals and retard oxidative processes [31]. Also, zinc plays an important role in DNA repair, protein synthesis and immune functioning [10, 31].

We observed a beneficial role of dietary iron on lung cancer risk, which was mainly driven by heme iron independent of red meat intake. Epidemiological results of total dietary iron and heme iron intake and lung cancer risk are mixed. Our results for dietary iron intake are in agreement with the findings from the NIH-AARP Diet and Health Study, a prospective study with 7052 lung cancer cases, which showed that the higher intake of dietary iron was associated with a significant reduced risk of lung cancer of 13 % [4]. A risk reduction ranging from 19 to 34 % for total iron intake was found in a case–control study with 1139 cases [16]. In another case control study, dietary iron intake was associated with an increased risk of lung cancer, whereas heme iron was associated with a decreased risk [5]. Similarly, in our study, heme iron was associated with a lower risk of lung cancer. However, a meta-analysis of three prospective studies on heme iron and lung cancer did not find any clear evidence for a beneficial role of heme iron [32]. Several factors may explain the discrepancies among reported results on dietary iron intake and lung cancer in epidemiological studies. First, the variation could be partly due to the complexity of assessing heme iron intake. A recent review on heme iron and lung cancer risk reported that from the total number of studies evaluating heme iron intake and lung cancer risk, only two studies used a specific food composition database [33, 34]. Second, different animal sources of heme iron such as poultry or fish have been associated with a decreased lung cancer risk [35, 36], whereas red meat is associated with an increased risk [33]. In the current investigation, we adjusted for red meat; therefore, it can be speculated that the observed beneficial role of heme iron may be due to other animal dietary sources. In addition, no association with heme iron was found when results were not adjusted for red meat. However, the exact mechanism for this observed protective effect of heme iron remains unclear. Last, inconsistent results between studies may be also due to different levels of dietary intake of minerals across studies, including iron intake [5, 18].

We found no role of dietary calcium, copper, magnesium and selenium intake on lung cancer risk. The association between these minerals and lung cancer has been investigated in few prospective studies with conflicting results. Our finding of no association of dietary magnesium intake and lung cancer risk is in agreement with the results of two previous cohort studies [18, 37]; however, others described an increased risk of lung cancer in subgroup analyses [4]. The relation between dietary copper intake and lung cancer risk has been rarely reported among cohort studies. Similar to our results, only one cohort study reported no association between dietary copper intake and lung cancer [4].

Evidence has shown that selenium is a potent antioxidant and may have anti-carcinogenic activity as well as selenium compounds are reported to induce toxicity and DNA damage [38]. However, our study and other previous prospective studies could not provide any support for a beneficial role of selenium on lung cancer risk [4, 39, 40]. Similarly, a selenium and lung cancer meta-analyses did not observe protective effect in studies assessing serum or dietary selenium but only in studies involving toenail selenium [41]. Also, a recent study showed a nonlinear association between selenium intake and esophageal cancer [30]. Furthermore, animal studies reported a different distribution of selenium between organs which suggest that benefit or risk differs from organ to organ [42]. In addition, data from previous studies show the influence of single-nucleotide polymorphisms in selenoprotein genes in lung cancer [43].

Our study is the largest cohort study to date with a long follow-up period (22 years) and which mutually investigate the association between dietary zinc, iron, calcium, copper, magnesium and selenium and lung cancer. In comparison with previous studies, our population was relatively homogenous with regard to external air quality, since all participants lived in the same district. Another strength is that we adjusted for a broad range of confounders taking into account also overall dietary quality and socioeconomic status (as a proxy also for occupational exposures). Furthermore, we were able to examine the associations of mineral intake with subtypes of lung cancer which did not show any difference. Nevertheless, there are several limitations that need to be taken into account. FFQs are known to have measurement errors. This mainly leads to biased results toward the null in estimating diet–disease risk. In addition, diet and supplement intake were assessed once at baseline and might not reflect long-term dietary exposures as would be expected from repeated assessments of diet during follow-up. However, it has been shown that although dietary intake may change due to development of diseases over time, using baseline diet instead of repeated measures of diet usually leads to an underestimation instead of an overestimation of the true association [44]. Furthermore, while dietary intake of selenium has been shown to alter plasma levels of selenium, it is not certain to which degree the intake of other minerals examined in this study reflects their status in the human body [45]. Also, we did not have biomarkers of mineral status in the body, which may be optimal to elucidate the role of minerals in the etiology of lung cancer. Moreover, due to limited number of lung cancer cases, we could not perform comprehensive subgroup analysis by smoking status to elucidate any potential interplay between mineral intake and smoking. Therefore, we cannot exclude residual confounding by smoking. We studied 6 different minerals in relation to lung cancer. However, it can be argued that this may lead to type I errors since we did not adjust the significance level for. Hence, cautious interpretation of *P* values is necessary.

In conclusion, our results suggest that a diet rich in zinc and iron may be associated with a decreased risk of lung cancer, whereas no evidence was found for other minerals. However, dietary mineral intake and the risk of lung cancer remain an understudied area of research, and further prospective studies are needed to better understand the possible role of minerals in lung carcinogenesis.

## Electronic supplementary material

Below is the link to the electronic supplementary material.

Supplementary material 1 (DOCX 53 kb)

## Abbreviations

BMI: Body mass index
DHD: Dutch healthy diet
FFQ: Food frequency questionnaire
ICD: International classification of disease
LC: Lung cancer
RS: Rotterdam Study
