# Supplementary material for: Dietary mineral intake and lung cancer risk: the Rotterdam Study
Source: Eur J Nutr. 2016 Apr 12;56(4):1637–46. doi: 10.1007/s00394-016-1210-4 (PMC5486639; doi:10.1007/s00394-016-1210-4)
Supplement: Supplementary file 1 — Supplementary material 1 (DOCX 53 kb) [file 394_2016_1210_MOESM1_ESM.docx]

**Dietary mineral intake and lung cancer risk: the Rotterdam Study**

Taulant Muka^1*^, Bledar Kraja^1,2,3*^, Rikje Ruiter^1,4^, Lies Lahousse^1, 5^, Catherine E. de Keyser^1, 6^, Albert Hofman^1^, Oscar H. Franco^1^, Guy Brusselle^5^, Bruno H. Stricker^1, 6^, Jessica C. Kiefte-de Jong^1, 7^

^*^These authors contributed equally

^1^Department of Epidemiology, Erasmus MC, Rotterdam, the Netherlands

^2^Department of Biomedical Sciences, Faculty of Medicine, University of Medicine, Tirana, Albania

^3^University Clinic of Gastrohepatology, University Hospital Center Mother Teresa, Tirana, Albania
^4^Department of Internal Medicine, Groene Hart Hospital, Gouda, the Netherlands

^5^Department of Respiratory Medicine, Ghent University Hospital, Ghent Belgium
^6^Health Care Inspectorate, the Hague, the Netherlands
^7^Leiden University College, The Hague, the Netherlands

**Corresponding author:** Professor Bruno H. Stricker PhD, Department of Epidemiology, Erasmus Medical Center**,** PO Box 2040**,** 3000 CA Rotterdam**,** the Netherlands. E-mail: b.stricker@erasmusmc.nl.

**Running title:** mineral intake and lung cancer

**Abbreviations:** BMI, body mass index; DHD, Dutch Healthy Diet; FFQ, food frequency questionnaire; ICD, International Classification of Disease; LC, lung cancer; RS, Rotterdam Study.

**Online Supplemental Material (OSM):** Six tables

|  | **Zinc** (mg/day) | **Iron** (mg/day) | **Magnesium** (mg/day) | **Selenium** (µg /day) | **Copper** (mg/day) | **Calcium** (mg/day) |
| --- | --- | --- | --- | --- | --- | --- |
| Zinc (mg/day) | 1 |  |  |  |  |  |
| Iron (mg/day) | 0.73 | 1 |  |  |  |  |
| Magnesium (mg/day) | 0.78 | 0.78 | 1 |  |  |  |
| Selenium (µg/day) | 0.61 | 0.47 | 0.58 | 1 |  |  |
| Copper (mg/day) | 0.15 | 0.24 | 0.46 | 0.43 | 1 |  |
| Calcium (mg/day) | 0.65 | 0.28 | 0.67 | 0.48 | 0.23 | 1 |

**Supplemental Table 1** Correlations coefficients between dietary minerals intake

**Supplemental Table 2** HRs of lung cancer by categories of dietary mineral intake in women

| **N=3210** | **Dietary Iron Intake** | | | *Continuous* | *Ptrend* |
| --- | --- | --- | --- | --- | --- |
|  | Tertile 1 | Tertile 2 | Tertile 3 |  |  |
| Cases, n | 28 | 29 | 26 |  |  |
| HR , 95% CI^a^ | 1.00 | 0.89 (0.49, 1.60) | 0.74 (0.34, 1.60) | 0.95 (0.79, 1.16) | 0.44 |
|  | **Dietary Copper Intake** | | | *Continuous* | *Ptrend* |
|  | Tertile 1 | Tertile 2 | Tertile 3 |  |  |
| Cases, n | 15 | 34 | 3 4 |  |  |
| HR , 95% CI^a^ | 1.00 | 2.19 (1.14, 4.20) | 2.13 (1.04, 4.33) | 1.72 (1.09, 2.73) | 0.06 |

HR, hazard ratio; CI, confidence interval

^a^ adjusted for age, alcohol intake (continuous), body mass index (continuous), smoking status(never smokers, former smokers <15 pack-years, former smoker ≥15 pack-year, current smoker <27.5 pack-years current smoker ≥27.5 pack-years), physical activity (continuous), Dutch healthy diet-index (continuous), dietary processed meat intake (continuous), dietary unprocessed red meat intake (continuous), total energy intake (continuous), hormone replacement therapy (yes vs. no), diabetes mellitus (yes vs. no), education status(low, intermediate, high), income status (low, intermediate, high), total energy adjusted sum of other minerals (excluding the mineral under investigation) (continuous) and family history of cancer (yes vs. no).

**Supplemental Table 3** HRs of lung cancer by categories of dietary mineral intake in men

HR, hazard ratio; CI, confidence interval

| **N=2222** | **Dietary Iron Intake** | | | *Continuous* | *Ptrend* |
| --- | --- | --- | --- | --- | --- |
|  | Tertile 1 | Tertile 2 | Tertile 3 |  |  |
| Cases, n | 57 | 36 | 35 |  |  |
| HR , 95% CI^a^ | 1.00 | 0.75 (0.47, 1.19) | 0.49 (0.28, 0.85) | 0.94 (0.84, 1.06) | 0.016 |
|  | **Dietary Copper Intake** | | | *Continuous* | *Ptrend* |
|  | Tertile 1 | Tertile 2 | Tertile 3 |  |  |
| Cases, n | 60 | 33 | 35 |  |  |
| HR , 95% CI^a^ | 1.00 | 1.10 (0.69, 1.74) | 0.96 (0.59, 1.55) | 0.97 (0.63, 1.49) | 0.90 |

^a^ adjusted for age, alcohol intake (continuous), body mass index (continuous), smoking status(never smokers, former smokers <15 pack-years, former smoker ≥15 pack-year, current smoker <27.5 pack-years current smoker ≥27.5 pack-years), physical activity (continuous), Dutch healthy diet-index (continuous), dietary processed meat intake (continuous), dietary unprocessed red meat intake (continuous), total energy intake (continuous), hormone replacement therapy (yes vs. no), diabetes mellitus (yes vs. no), education status(low, intermediate, high), income status(low, intermediate, high), total energy adjusted sum of other minerals (excluding the mineral under investigation) (continuous) and family history of cancer (yes vs. no).

| **N=5270** | **Dietary Zinc Intake** | | | *Continuous* | *Ptrend* |
| --- | --- | --- | --- | --- | --- |
|  | Tertile 1 | Tertile 2 | Tertile 3 |  |  |
| Cases, n | 70 | 70 | 47 |  |  |
| HR, 95% CI^a^ | 1.00 | 1.07 (0.73, 1.56) | 0.60  (0.36, 1.00) | 0.88 (0.79, 0.98) | 0.07 |
|  | **Dietary Iron Intake** | | | *Continuous* | *Ptrend* |
|  | Tertile 1 | Tertile 2 | Tertile 3 |  |  |
| Cases, n | 74 | 58 | 55 |  |  |
| HR, 95% CI^a^ | 1.00 | 0.79 (0.53, 1.16) | 0.58 (0.35, 0.95) | 0.93 (0.84, 1.04) | 0.031 |
|  |  | **Dietary Magnesium Intake** | | *Continuous* | *Ptrend* |
|  | Tertile 1 | Tertile 2 | Tertile 3 |  |  |
| Cases, n | 75 | 57 | 55 |  |  |
| HR , 95% CI^a^ | 1.00 | 0.85 (0.59, 1.23) | 0.80  (0.51, 1.24) | 0.997 (0.994, 1.01) | 0.30 |
|  |  | **Dietary Selenium Intake** | | *Continuous* | *Ptrend* |
|  | Tertile 1 | Tertile 2 | Tertile 3 |  |  |
| Cases, n | 61 | 54 | 73 |  |  |
| HR , 95% CI^a^ | 1.00 | 1.04 (0.71-1.52) | 1.44  (0.98-2.11) | 1.01 (0.998-1.03) | 0.056 |
|  |  | **Dietary Copper Intake** | | *Continuous* | *Ptrend* |
|  | Tertile 1 | Tertile 2 | Tertile 3 |  |  |
| Cases, n | 62 | 65 | 60 |  |  |
| HR , 95% CI^a^ | 1.00 | 1.52 (1.04, 2.22) | 1.26 (0.83, 1.91) | 1.25 (0.93, 1.69) | 0.28 |
|  |  | **Dietary Calcium Intake** | | *Continuous* | *Ptrend* |
|  | Tertile 1 | Tertile 2 | Tertile 3 |  |  |
| Cases, n | 78 | 58 | 51 |  |  |
| HR , 95% CI^a^ | 1.00 | 0.88 (0.58, 1.34) | 0.71  (0.36, 1.39) | 0.994  (0.882, 1.12) | 0.33 |
|  |  | **Dietary Heme-iron Intake** | | *Continuous* | *Ptrend* |
|  | Tertile 1 | Tertile 2 | Tertile 3 |  |  |
| Cases, n | 67 | 65 | 55 |  |  |
| HR, 95% CI^a^ | 1.00 | 0.89 (0.62, 1.27) | 0.57 (0.3, 0.89) | 0.83 (0.67, 1.03) | 0.013 |
|  |  | **Dietary non-Heme-iron Intake** | | *Continuous* | *Ptrend* |
|  | Tertile 1 | Tertile 2 | Tertile 3 |  |  |
| Cases, n | 68 | 64 | 55 |  |  |
| HR , 95% CI^a^ | 1.00 | 1.16 (0.79, 1.71) | 0.98 (0.59, 1.62) | 0.98 (0.87, 1.10) | 0.996 |

**Supplemental Table 4** Multivariable HRs of lung cancer by categories of dietary mineral intake after excluding lung cancer within the first two years of follow-up

HR, hazard ratio; CI, confidence interval
^a^ adjusted for age, sex, alcohol intake (continuous), body mass index (continuous), smoking status(never smokers, former smokers <15 pack-years, former smoker ≥15 pack-year, current smoker <27.5 pack-years current smoker ≥27.5 pack-years), physical activity (continuous), Dutch healthy diet-index (continuous), dietary processed meat intake (continuous), dietary unprocessed red meat intake (continuous), total energy intake (continuous), hormone replacement therapy (yes vs. no), diabetes mellitus (yes vs. no), education status(low, intermediate, high), income status (low, intermediate, high), total energy adjusted sum of other minerals (excluding the mineral under investigation) (continuous) and family history of cancer (yes vs. no).

| **N=5435** | **Dietary Zinc Intake** | | | *Continuous* | *Ptrend* |
| --- | --- | --- | --- | --- | --- |
|  | Tertile 1 | Tertile 2 | Tertile 3 |  |  |
| Cases, n | 53 | 56 | 33 |  |  |
| Model 1 HR, 95% CI^a^ | 1.00 | 1.08 (0.74, 1.57) | 0.58  (0.37, 0.89) | 0.90  (0.83, 0.98) | 0.017 |
| Model 2 HR , 95% CI^b^ | 1.00 | 1.24 (0.81, 1.90) | 0.61  (0.34, 0.11) | 0.90  (0.79, 1.01) | 0.15 |
|  | **Dietary Iron Intake** | | | *Continuous* | *Ptrend* |
|  | Tertile 1 | Tertile 2 | Tertile 3 |  |  |
| Cases, n | 57 | 4 7 | 38 |  |  |
| Model 1 HR , 95% CI^a^ | 1.00 | 0.79 (0.54, 1.17) | 0.54  (0.36, 0.83) | 0.95  (0.86, 1.00) | 0.004 |
| Model 2 HR , 95% CI^b^ | 1.00 | 0.88 (0.57, 1.36) | 0.53  (0.30, 0.93) | 0.95  (0.85, 1.06) | 0.029 |
|  |  | **Dietary Magnesium Intake** | | *Continuous* | *Ptrend* |
|  | Tertile 1 | Tertile 2 | Tertile 3 |  |  |
| Cases, n | 61 | 40 | 41 |  |  |
| Model 1 HR , 95% CI^a^ | 1.00 | 0.67 (0.45, 0.995) | 0.62  (0.41, 0.92) | 0.997  (0.994, 1.00) | 0.016 |
| Model 2 HR , 95% CI^b^ | 1.00 | 0.77 (0.50, 1.18) | 0.74  (0.45, 1.22) | 0.998  (0.994, 1.002) | 0.22 |
|  |  | **Dietary Selenium Intake** | | *Continuous* | *Ptrend* |
|  | Tertile 1 | Tertile 2 | Tertile 3 |  |  |
| Cases,n | 48 | 43 | 51 |  |  |
| Model 1 HR , 95% CI^a^ | 1.00 | 0.97 (0.64, 1.47) | 1.11  (0.74, 1.65) | 1.01  (0.99, 1.03) | 0.61 |
| Model 2 HR , 95% CI^b^ | 1.00 | 1.10  (0.71, 1.69) | 1.30  (0.84, 2.01) | 1.01  (0.99, 1.03) | 0.24 |
|  |  | **Dietary Copper Intake** | | *Continuous* | *Ptrend* |
|  | Tertile 1 | Tertile 2 | Tertile 3 |  |  |
| Cases, n | 48 | 47 | 47 |  |  |
| Model 1 HR , 95% CI^a^ | 1.00 | 1.09 (0.72, 1.63) | 1.06  (0.71, 1.59) | 1.16  (0.95, 1.41) | 0.78 |
| Model 2 HR , 95% CI^b^ | 1.00 | 1.57 (1.01, 2.44) | 1.39  (0.87, 2.23) | 1.11  (0.80, 1.53) | 0.16 |
|  |  | **Dietary Calcium Intake** | | *Continuous* | *Ptrend* |
|  | Tertile 1 | Tertile 2 | Tertile 3 |  |  |
| Cases, n | 57 | 5 | 35 |  |  |
| Model 1 HR , 95% CI^a^ | 1.00 | 1.01 (0.69, 1.48) | 0.73  (0.48, 1.12) | 1.000  (0.999, 1.000) | 0.17 |
| Model 2 HR , 95% CI^b^ | 1.00 | 1.12 (0.69, 1.82) | 0.74  (0.33, 1.65) | 1.02  (0.90, 1.16) | 0.61 |

**Supplemental Table 5** HRs of non-small cell lung cancer by categories of dietary mineral intake*

HR, hazard ratio; CI, confidence interval

^*^ Lung cancer cases were classified as small cell lung cancer (SCLC) and non-small cell lung cancer (NSCLC). In addition, NSCLC were sub-classified according to three main subtypes of the World Health Organization (WHO)/International Association for the Study of Lung Cancer (IASLC) histologic classification of NSCLCs into adenocarcinoma, large cell carcinoma and squamous cell carcinoma.

^a^ adjusted for age and sex.
^b^ adjusted for age, sex, alcohol intake(continuous), body mass index (continuous), smoking status(never smokers, former smokers <15 pack-years, former smoker ≥15 pack-year, current smoker <27.5 pack-years current smoker ≥27.5 pack-years), physical activity (continuous), Dutch healthy diet-index (continuous), dietary processed meat intake(continuous), dietary unprocessed red meat intake (continuous), total energy intake(continuous), hormone replacement therapy (yes vs. no), diabetes mellitus (yes vs. no), education status (low, intermediate, high), income status(low, intermediate, high), total energy adjusted sum of other minerals (excluding the mineral under investigation) (continuous) and family history of cancer (yes vs. no).

**Supplemental Table 6** HRs of lung cancer other than non-small cell lung cancer by categories of dietary mineral intake*

| **N=5435** | **Dietary Zinc Intake** | | | *Continuous* | *Ptrend* |
| --- | --- | --- | --- | --- | --- |
|  | Tertile 1 | Tertile 2 | Tertile 3 |  |  |
| Cases, n | 30 | 21 | 18 |  |  |
| Model 1 HR, 95% CI^a^ | 1.00 | 0.69 (0.39, 1.20) | 0.57  (0.31, 1.02) | 0.89  (0.78, 1.00) | 0.05 |
| Model 2 HR , 95% CI^b^ | 1.00 | 0.69 (0.37, 1.30) | 0.52  (0.23, 1.22) | 0.86  (0.71, 1.04) | 0.13 |
|  | **Dietary Iron Intake** | | | *Continuous* | *Ptrend* |
|  | Tertile 1 | Tertile 2 | Tertile 3 |  |  |
| Cases, n | 28 | 18 | 23 |  |  |
| Model 1 HR , 95% CI^a^ | 1.00 | 0.60 (0.33, 1.08) | 0.73  (0.41, 1.28) | 0.95  (0.85, 1.06) | 0.26 |
| Model 2 HR , 95% CI^b^ | 1.00 | 0.65 (0.34, 1.26) | 0.70  (0.31, 1.56) | 0.94  (0.79, 1.12) | 0.36 |
|  |  | **Dietary Magnesium Intake** | | *Continuous* | *Ptrend* |
|  | Tertile 1 | Tertile 2 | Tertile 3 |  |  |
| Cases, n | 27 | 20 | 22 |  |  |
| Model 1 HR , 95% CI^a^ | 1.00 | 0.71 (0.40, 1.27) | 0.75  (0.42, 1.32) | 0.997 (0.992, 1.001) | 0.31 |
| Model 2 HR , 95% CI^b^ | 1.00 | 0.89 (0.48, 1.65) | 1.12  (0.54, 2.29) | 0.998 (0.993, 1.004) | 0.80 |
|  |  | **Dietary Selenium Intake** | | *Continuous* | *Ptrend* |
|  | Tertile 1 | Tertile 2 | Tertile 3 |  |  |
| Cases, n | 22 | 19 | 28 |  |  |
| Model 1 HR , 95% CI^a^ | 1.00 | 0.86 (0.47, 1.60) | 1.27  (0.73, 2.23) | 1.01  (0.98, 1.04) | 0.38 |
| Model 2 HR , 95% CI^b^ | 1.00 | 1.003  (0.53, 1.91) | 1.65  (0.88, 3.09) | 1.02  (0.99, 1.04) | 0.11 |
|  |  | **Dietary Copper Intake** | | *Continuous* | *Ptrend* |
|  | Tertile 1 | Tertile 2 | Tertile 3 |  |  |
| Cases, n | 27 | 20 | 22 |  |  |
| Model 1 HR , 95% CI^a^ | 1.00 | 0.73 (0.41-1.31) | 0.80  (0.45-1.41) | 1.20 (0.91-1.57) | 0.43 |
| Model 2 HR , 95% CI^b^ | 1.00 | 0.96 (0.51, 0.90) | 0.97  (0.49, 1.90) | 1.44  (0.86, 2.40) | 0.92 |
|  |  | **Dietary Calcium Intake** | | *Continuous* | *Ptrend* |
|  | Tertile 1 | Tertile 2 | Tertile 3 |  |  |
| Cases, n | 33 | 14 | 22 |  |  |
| Model 1 HR , 95% CI^a^ | 1.00 | 0.43 (0.23, 0.81) | 0.68  (0.39, 1.18) | 1.000  (0.999, 1.000) | 0.14 |
| Model 2 HR , 95% CI^b^ | 1.00 | 0.49 (0.23, 1.04) | 0.78  (0.27, 2.26) | 1.01  (0.82, 1.22) | 0.45 |

HR, hazard ratio; CI, confidence interval

*Lung cancer cases were classified as small cell lung cancer (SCLC) and non-small cell lung cancer (NSCLC). In addition, NSCLC were sub-classified according to three main subtypes of the World Health Organization (WHO)/International Association for the Study of Lung Cancer (IASLC) histologic classification of NSCLCs into adenocarcinoma, large cell carcinoma and squamous cell carcinoma.
^a^ adjusted for age and sex.
^b^ adjusted for age, sex, alcohol intake (continuous), body mass index (continuous), smoking status (never smokers, former smokers <15 pack-years, former smoker ≥15 pack-year, current smoker <27.5 pack-years current smoker ≥27.5 pack-years), physical activity (continuous), Dutch healthy diet-index (continuous), dietary processed meat intake (continuous), dietary unprocessed red meat intake (continuous), total energy intake (continuous), hormone replacement therapy (yes vs. no), diabetes mellitus (yes vs. no), education status(low, intermediate, high), income status(low, intermediate, high), total energy adjusted sum of other minerals (excluding the mineral under investigation) (continuous) and family history of cancer (yes vs. no).
